# Single-Cell Proteomic Characterization of Drug-Resistant Prostate Cancer Cells Reveals Molecular Signatures Associated with Morphological Changes

**DOI:** 10.1016/j.mcpro.2025.100949

**Published:** 2025-03-14

**Authors:** Jongmin Woo, Michael Loycano, Md Amanullah, Jiang Qian, Sarah R. Amend, Kenneth J. Pienta, Hui Zhang

**Affiliations:** 1 Department of Pathology, Johns Hopkins University School of Medicine, Baltimore, Maryland, USA; 2 Department of Urology, Johns Hopkins University School of Medicine, Baltimore, Maryland, USA; 3 Cancer Ecology Center, James Buchanan Brady Urological Institute, Johns Hopkins University School of Medicine, Baltimore, Maryland, USA; 4 Department of Ophthalmology, Johns Hopkins University School of Medicine, Baltimore, Maryland, USA; 5 Department of Oncology, Johns Hopkins University School of Medicine, Baltimore, Maryland, USA

**Keywords:** prostate cancer cell line, drug resistant, single-cell proteomics, molecular signature

## Abstract

This study delves into the proteomic intricacies of drug-resistant cells (DRCs) within prostate cancer, which are known for their pivotal roles in therapeutic resistance, relapse, and metastasis. Utilizing single-cell proteomics (SCP) with an optimized high-throughput data-independent acquisition (DIA) approach with the throughput of 60 sample per day, we characterized the proteomic landscape of DRCs in comparison to parental PC3 cells. This DIA method allowed for robust and reproducible protein quantification at the single-cell level, enabling the identification and quantification of over 1300 proteins per cell on average. Distinct proteomic sub-clusters within the DRC population were identified, closely linked to variations in cell size. The study uncovered novel protein signatures, including the regulation of proteins critical for cell adhesion and metabolic processes, as well as the upregulation of surface proteins and transcription factors pivotal for cancer progression. Furthermore, by conducting single-cell RNA-seq (scRNA-seq) analysis, we identified six upregulated and 10 downregulated genes consistently altered in drug-treated cells across both SCP and scRNA-seq platforms. These findings underscore the heterogeneity of DRCs and their unique molecular signatures, providing valuable insights into their biological behavior and potential therapeutic targets.

## Highlights

**This study employs…**
• Single-cell proteomics profiles prostate cancer drug-resistant cell heterogeneity.
• Optimized cellenONE protocol enables isolation of large polyaneuploid DRCs.
• Identified three proteomic DRC subtypes linked to size and functional traits.
• Revealed key surface markers and TFs associated with invasiveness in DRCs.
• Provides targets and insights into resistance, relapse, and metastatic risk.

## Graphical Abstract

**Figure:**
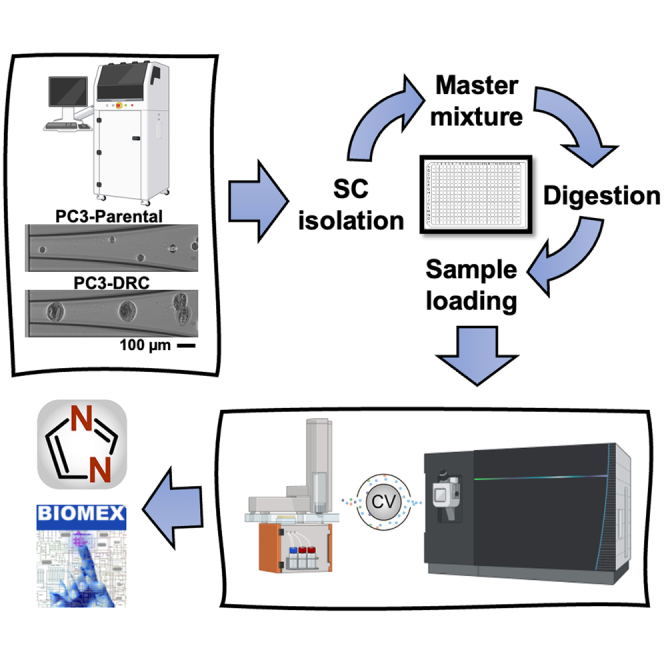

## In Brief

This study pioneers the use of single-cell proteomics to uncover molecular signatures associated with drug-resistant prostate cancer cells (DRCs), identifying key protein pathways that drive their adaptability, resistance, and metastasis. By employing advanced DIA-MS and integrating transcriptomics data, the work reveals unique proteomic sub-clusters linked to cell morphology and size, offering new therapeutic insights into these aggressive cancer cell populations.

Cancer is a heterogeneous disease characterized by diverse cell populations with distinct genetic and phenotypic profiles (1, 2). Among these, drug-resistant cells (DRCs) have garnered significant attention due to their association with therapeutic resistance, disease relapse, and metastasis (3, 4). DRCs are characterized by their large size and complex karyotypes, which contribute to their adaptability and survival under therapeutic stress (5, 6). DRCs have been identified in various cancer types, including breast, colorectal, and ovarian cancers, where they correlate with poor prognosis and survival (7, 8, 9, 10, 11, 12). These cells often arise due to oncogenic and therapeutic stress, leading to genomic instability and the formation of cells with multiple nuclei and enlarged size (13, 14). Recent advancements in single-cell technologies have enabled detailed molecular characterizations of these rare cell populations (11). While single-cell RNA-seq (scRNA-seq) has provided valuable insights into the transcriptional landscapes of DRCs, proteomics offers a complementary approach by directly measuring the functional protein molecules that drive cellular behavior (15, 16). Therefore, this study focuses on the proteomic characterization of DRCs of prostate cancer cells at the single-cell level, aiming to uncover the proteomic alterations that underpin their unique biological properties.

Single-cell proteomics, particularly when combined with high-throughput methodologies such as Data Independent Acquisition Mass Spectrometry (DIA-MS) (17) and Liquid Chromatography with Field Asymmetric Ion Mobility Spectrometry Mass Spectrometry (LC-FAIMS-MS) (18), allows for an in-depth analysis of the proteomic landscape of these cells. By employing state-of-the-art single-cell proteomics, we not only capture the extensive proteomic repertoire of these cells but also benchmark the reproducibility and quantification capabilities for single-cell proteomic characterization of these cells. This benchmarking is critical, as it ensures the reliability of data especially when comparing the complex proteomes of distinct cell populations like DRCs and their parental cell lines. In addition, isolating DRCs poses significant challenges due to their size (19, 20). In this study, we employed the cellenONE instrument for cell isolation, addressing size-related issues by diluting and percolating cells through a strainer to ensure accurate sampling. This study marks the first detailed molecular characterization of DRCs at the single-cell level, providing novel insights into their biology and potential therapeutic vulnerabilities. By integrating these findings with scRNA-seq, we aim to contribute to the growing body of knowledge on DRCs and highlight the potential of single-cell proteomics in advancing clinical cancer research. Our work underscores the importance of studying these enigmatic cells to develop more effective therapeutic strategies against prostate cancer

## Method

### Cell Culture and Drug-Resistance Cell Induction

**Fig. 1:**
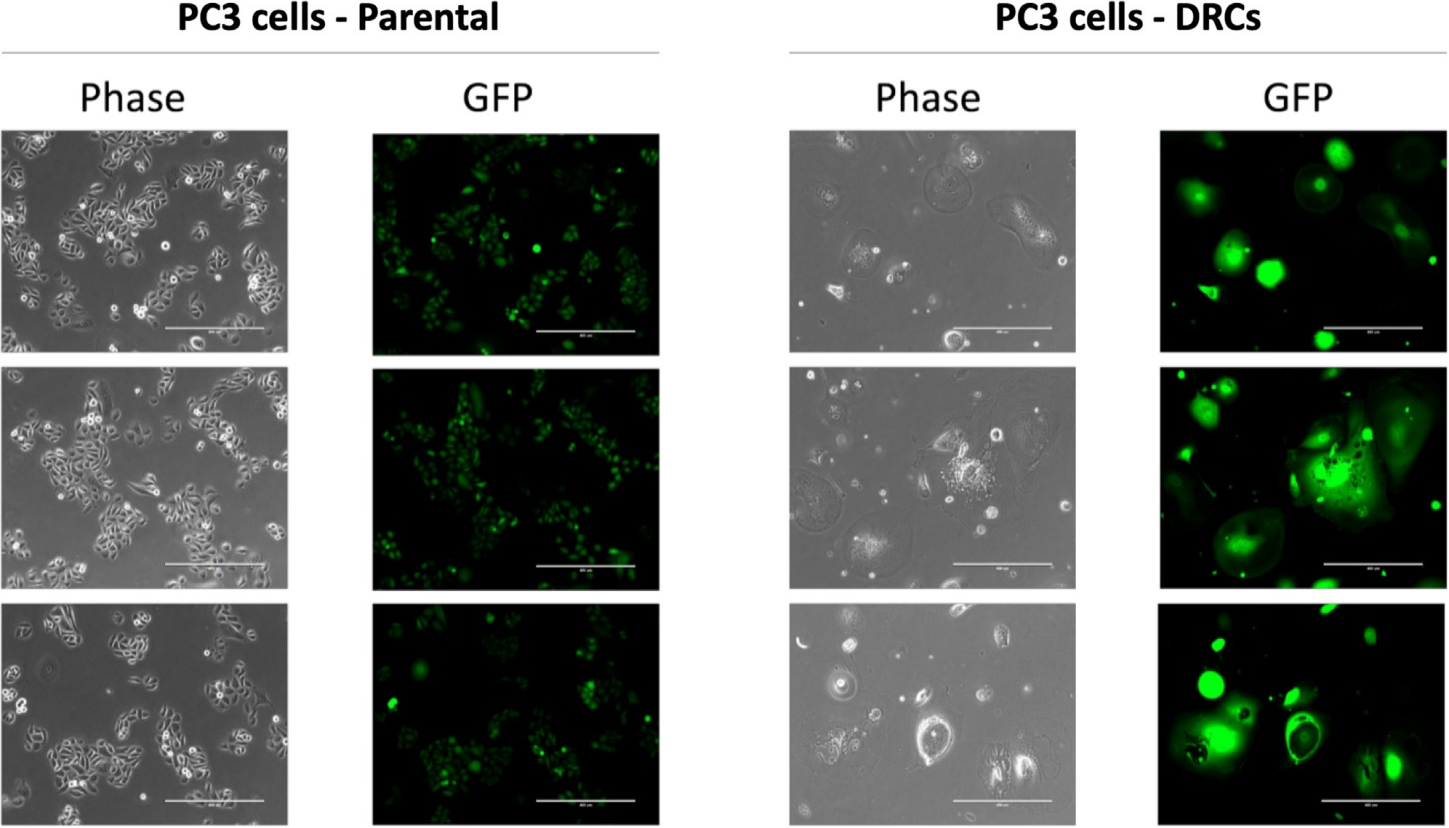
**Morphological and fluorescence analysis of parental PC3 cells and drug-resistant cells (DRCs).** Each column represents triplicate images taken from different areas within the same dish. The *left* panels in each column show bright-field images, highlighting differences in cell morphology and size between parental PC3 cells and DRCs. The *right* panels display fluorescence images, confirming consistent GFP expression. Scale bar = 400 μm.

Cell culture experiments were conducted using the PC3-luciferase-GFP prostate cancer cell line (ATCC and edited by the UMich Cell Line Core and Pienta’s group at JHU) (5, 17, 21, 22, 23, 24). The cells were cultured in RPMI 1640 medium with L-glutamine and phenol red (Gibco), supplemented with 10% Premium Grade Fetal Bovine Serum (Avantor Seradigm) and 1% Penicillin-Streptomycin (5000 U/ml; Gibco), and maintained at 37°C in a 5% CO2 incubator. Authentication and *mycoplasma* contamination testing of the PC3 cells were performed biannually by Genetica. Cells were seeded at a density of 6,000,000 cells per 500cm2 dish (Corning). Twenty-four hours post-seeding, the cells were treated with 6 μM (GI50) cisplatin, dissolved in PBS with 140 mM NaCl, for 72 h (Millipore Sigma). After the treatment, the medium was replaced with fresh medium, and the cells were cultured for an additional 10 days, at which point they were considered definitive drug-resistant cells (DRCs). The medium was refreshed every 4 days. TrypLE Express Enzyme without phenol red was routinely used for cell dissociation and plates were scraped using a cell scraper. All centrifugation was done at 200×*g* for 10 min. Cells were resuspended in PBS for transport. Images were captured using the EVOS M7000 and EVOS FL Auto microscopes (ThermoFisher, Fig. 1). Both PC3 parental and DRCs were immediately transported on ice for cell isolation.

### Single-Cell Isolation for Proteomics and RNA-Seq Studies

Single cells were isolated using the cellenONE instrument, which enables precise manipulation of individual cells via real-time imaging that assesses cell size. PC3 parental cells were first diluted in PBS to a concentration of 200 cells/μl and isolated based on a cell size range of 22 to 25 μm in diameter. For the isolation of large DRCs, the concentration was further diluted to ∼50 cells/μl to prevent clogging the cell sorting nozzle of the cellenONE system during the cell sorting. This dilution allowed for the isolation of DRCs up to ∼102 μm in diameter, even though the cells were pre-filtered through a 70 μm cell strainer. The cellenONE system was equipped with a large-size Piezo Dispense Capillary (PDC, Cellenion, P-20-CL) with a drop volume of 450 to 600 pL and an inner dimension of ∼80 μm. Although the PDC size typically limits the isolation of cells larger than 80 μm, the inherent elasticity of DRCs enabled them to deform and pass through the nozzle, facilitating their isolation. These adjustments were critical for handling the larger size and heterogeneous nature of the DRC population. Each single cell was dispensed into a well of a 384-well plate containing 1 μl of pre-added lysis buffer, prepared for the subsequent digestion procedure. During the isolation process, images of all isolated cells were captured, including measurements of cell diameter and bright-field intensity, for further analysis.

### Single-Cell Sample Preparation for Proteomic Analysis

Isolated cells underwent lysis, protein extraction, and digestion, as the on-plate sample preparation (25). In brief, 1 μl of lysis buffer—comprising 0.1% DDM, 0.02% LMNG, and 5 mM TCEP in a 100 mM TEAB buffer—was added to each well of a 384-well plate prior to cell isolation. After cell deposition, the plate was centrifuged briefly at 1000 x g for 5 min. For the digestion step, 0.5 μl of a trypsin/LysC mixture (5 ng total) was added to each sample. The plate was thoroughly sealed with an Adhesive Plate Seal (LabViva, Cat# 186006336) to prevent evaporation and incubated overnight at 37°C with a heated lid (Benchmark, H5100-HCT and H5100-HL) to maintain a stable temperature and reduce evaporation. The resulting peptide samples were acidified and stored at −80°C until ready for LC-MS analysis. For loading onto the Evotips, which had been washed and activated according to the manufacturer’s instructions, peptides were first thoroughly washed in 10 μl of 0.1% formic acid before being transferred to the Evotips.

### Bulk Sample Preparation for Spectral Library Generation

A total of half million cells, either from PC3 parental lines or DRC, were collected and lysed in 5% SDS. Protein digestion was performed using the S-Trap micro (PROTIFI, Fairport, NY) following the manufacturer’s instructions. Briefly, protein concentration was measured after lysis, and 100 μg of protein was reduced with 5 mM TCEP and alkylated with 20 mM IAA. Subsequently, 2.5% phosphoric acid and binding buffer (90% ethanol in 100 mM TEAB) were added to the mixture before loading it onto the S-Trap column placed in a 1.7 ml receiver tube. After three washes with wash buffer, the bound proteins on the column were digested overnight with LysC and Trypsin. Finally, 10 ng of the digested peptides were used for spectral library generation.

### Serial Diluted Peptide Sample Preparation

NCI-7 cells were used as reference materials (26). Peptides from NCI-7 cells were prepared following the CPTAC protocol. Briefly, 1 x 10^∧^6 cells were lysed via centrifugation at 10,000 x g for 10 min. The extracted proteins were denatured using 8M urea, reduced with 10 mM DTT, and alkylated with 20 mM IAA. The proteins were then digested with trypsin and the resulting peptides were serially diluted across nine dilution points, ranging from 400 ng to 125 pg.

### Experimental Design and Statistical Rationale

To ensure reproducibility and reliability, technical replicates were included during LC-MS analysis. Specifically, three technical replicates were conducted for both bulk peptide samples used for spectral library generation and for diluted peptide samples toward the single cell or sub-cellular peptide levels. In the single-cell proteomics study, we analyzed a total of 144 single cells, comprising 72 drug-resistant cells (DRCs) and 72 parental PC3 cells. For the single-cell RNA sequencing (scRNA-seq) analysis, a total of 27 single cells were analyzed, including 11 DRCs and 16 parental PC3 cells. The scRNA-seq experiments were conducted at the JHU Core Facility, where the number of analyzed cells was limited due to resources.

Statistical significance was assessed using rigorous methods to ensure the reliability of the results. Quantitative values were log2-transformed, and protein groups were identified if quantitative values were present. For protein quantification, proteins were filtered by quantified proteins if quantified at least 70% of the samples in each experimental group contained valid measurements. Missing values were then imputed using the K-nearest neighbor (KNN) algorithm with a neighbor number of 15 to minimize bias (27). A two-sample *t* test with a significance threshold of *p*-value <0.05 was applied to identify differentially expressed proteins. This comprehensive approach ensured that the number of biological and technical replicates was sufficient to detect statistically significant differences in proteomic profiles across experimental conditions.

### Proteomic Analysis Using LC-MS Instruments

Proteomic analyses were performed by DIA-MS using two distinct instruments paired with the Evosep LC: the timsTOF HT (Bruker) and the Orbitrap Ascend (Thermo). The diluted peptide samples were separated in the Evosep using short gradients, allowing for high-throughput analysis of up to 100 samples per day (SPD) utilizing an analytical column (PepSep, 8 cm × 100 μm x 3 μm). For the trapped ion mobility spectrometry (TIMS) on the timsTOF, we set a mobility range of 0.70 to 1.45 V s/cm^2^ and a mass range of 100 to 1700 m/z. An accumulation time of 166 ms was used with high-sensitivity detection enabled when peptide quantities were below 50 ng. In addition, collision energy settings are 20 eV at 0.60 V s/cm2 and 59 eV at 1.60 V s/cm2. For DIA-PASEF, mass width was set as 25 Da ranging from 338.6 Da to 1338.6 Da, resulting in a total of 40 mass steps per cycle and 1.72 s estimated cycle time. For higher input samples (>50 ng peptides), we reduced the accumulation time to 85 ms and disabled high-sensitivity detection. The Orbitrap Ascend instrument, equipped with Field Asymmetric Ion Mobility Spectrometry (FAIMS), was configured with a compensation voltage of −55 V, a mass range of 380 to 980 m/z, an MS2 scan range of 145 to 1450 m/z, a resolution of 30,000, and an Automatic Gain Control (AGC) target of 2.5 × 10E5. For low-input samples (<50 ng), acquisition parameters included a 35 m/z isolation window with a 0.5 m/z overlap (yielding 18 scan events in total) and a maximum injection time (IT) of 166 ms. For high-input samples, we applied a narrower isolation window of 18 m/z with a 0.5 m/z overlap (totaling 34 scan events) and an IT of 59 ms.

In the single-cell analysis, including spectra library generation, using the Orbitrap Ascend coupled with Evosep LC and FAIMS (−55 CV), samples were separated utilizing a throughput of 60 SPD with the same analytical column. Parameters remained consistent with those listed above, but the mass range was adjusted to 380 to 780 m/z and the isolation window was narrowed to 25 m/z with 16 number of scan events to optimize protein identification and quantification. Ions were accumulated during the IT of 180 ms.

### Data Analysis for scProteomics

Proteomic raw data for instrument performance assessment using timsTOF HT and FAIMS-Ascend were processed using Spectronaut (version 18.3, FASTA database downloaded 09/15/2023, UniprotKB reviewed, 20,426 entries) with the direct-DIA approach to benchmark the two datasets, while single-cell data obtained from FAIMS-Ascend were analyzed using DIA-NN (version 1.8.0), employing a library-based database search. For both tools, acetylation at the protein N-terminus (Acetyl [N-term]) and oxidation of methionine residues (Oxidation [M]) were included as variable modifications, with a maximum of three variable modifications allowed per peptide. In Spectronaut, the mass tolerance for precursor and fragment ions was set to dynamic, allowing the software to infer appropriate tolerances based on the data. False discovery rates (FDR) for both peptide and protein levels were controlled at 1% in both Spectronaut and DIA-NN. Raw data from timsTOF HT and Orbitrap Ascend were processed separately using Spectronaut to ensure accurate peptide and protein identification. Due to the inherent differences in fragmentation patterns between the two instruments—timsTOF HT using trapped ion mobility separation (tims) and PASEF-DIA acquisition, and Orbitrap Ascend using FAIMS-DIA acquisition—a combined search could introduce biases or inconsistencies in the identification and quantification of peptides. To avoid such potential interferences, data were searched independently for each dataset. Quantification results were then analyzed separately and used for comparative evaluation of the two proteomic platforms.

The spectral library for DIA data analysis was generated using FragPipe (version 21.1, FASTA database downloaded 09/15/2023, UniprotKB reviewed, 20,426 entries) with easyPQP (version 0.1.50). Bulk peptide samples from PC3 parental and DRC cells were processed for spectral library generation. The precursor mass tolerance and fragment mass tolerance were set at 20 ppm. The number of missed cleavages was set to 1. Fixed and variable modifications were consistent with those used in downstream tools. The retention time (RT) calibration was performed using calibration indexed retention Time (ciRT) as a reference, and b and y ion spectra were generated with 0.02 Da UniMod annotation and 15 ppm fragment annotation. This library provided comprehensive coverage, containing 7095 precursors and 2034 proteins. These precursors and proteins were selected based on high confidence scores and consistent detection across multiple replicates. The resulting library was used for library-based database searching of single-cell data with DIA-NN. In DIA-NN, both MS1 and MS2 accuracies were set at 10 ppm, with a scan window of 3. Features such as isotopologues, match-between-runs, and unrelated runs were enabled, while cross-normalization was disabled. Down-stream data analysis including statistics and bioinformatics was performed using Perseus (Ver. 1.6.15) (28), BIOMEX (Ver. 1.0.15) (29), and String DB for the Reactome Pathway enrichment analysis (Ver. 12.0) (30). Multivariate analyses, including Principal Component Analysis (PCA) and Uniform Manifold Approximation and Projection (UMAP), were performed to evaluate overall proteomic variability and identify clustering patterns. Correlation and bioinformatics analyses were performed to investigate relationships between cell volume and proteome abundance.

### Transcriptomics Analysis

RNA from single cells in lysis buffer was immediately processed for reverse transcription. RNA quality was assessed using an Agilent Bioanalyzer (Agilent Technologies). mRNA reverse transcription was performed using the SMART-Seq v4 Oligonucleotide technology, which employs oligo(dT) priming to synthesize full-length cDNA. The reaction was carried out at 42°C for 90 min, followed by enzyme inactivation at 70°C for 10 min. The first-strand cDNA synthesis was optimized with the inclusion of locked nucleic acids (LNA) to enhance template-switching efficiency. Amplification of the first-strand cDNA was performed using Long-Distance PCR (LD-PCR) with SeqAmp DNA polymerase. The number of PCR cycles was adjusted according to the RNA input, with 17 cycles used for 10 pg of RNA. The amplified cDNA was then purified using AMPure XP beads (Beckman Coulter). cDNA was quantified using the Qubit dsDNA HS Assay (Thermo Fisher Scientific) and assessed for quality using an Agilent Bioanalyzer to confirm the presence of high-quality, full-length cDNA. For sequencing, cDNA libraries were prepared using Takara Bio’s SMART-Seq Library Prep Kit, which utilizes stem-loop adapters for enzymatic fragmentation and indexing. The libraries were further amplified through an additional 12 to 16 PCR cycles, depending on the input cDNA concentration. After amplification, samples were pooled, purified with AMPure XP beads, and validated for library-size distribution using an Agilent Bioanalyzer. Library quantification was conducted via qPCR before sequencing on an Illumina platform, utilizing paired-end 150 bp reads.

### Sequence Alignment and Gene Expression Quantification

FastQC (v0.12.1) tool was used to assess the quality of raw sequencing data. The quality of sequencing data demonstrated high-quality metrics, with the majority of reads exhibiting Phred quality scores above 30 across all bases. Key quality indicators, such as base sequence quality, GC content, and adapter contamination, were within acceptable ranges, indicating that the sequencing data was of good quality. Paired-end sequencing reads were then mapped to the reference genome using the STAR (2.7.11 b) Aligner following the standard pipeline (31). To obtain accurate and high-speed quantification of read counts, featureCounts function from the Subread package (v2.0.6) was used based on the GRCm39.111 GTF annotation data (32). The human reference genome (GRCh38) and gene annotation (GRCh38.111) GTF data were downloaded from Ensemble database. To reduce noise from lowly expressed genes, genes with less than 1 CPM value across the samples were then filtered, leaving 25,651 genes for subsequent analysis. Following this filtering step, only genes with reliable expression patterns were retained for further analysis. Similar to our analysis of the SCP data, quantitative values were log2-transformed. Protein groups were considered identified if quantitative values were present and considered quantified if at least 70% of the samples in each group contained valid quantitative measurements. For the comparison between SCP and scRNA-seq data, both datasets were normalized using width adjustment, aligning the median to zero across all quantitative protein groups. Statistical analysis was performed using a *t* test with a *p*-value threshold of <0.05.

## Results

### Evaluation of Analytical Performances of DIA-MS in Proteomic Analysis of Single-Cell Level Peptides With Two Proteomic Platforms

**Fig. 2:**
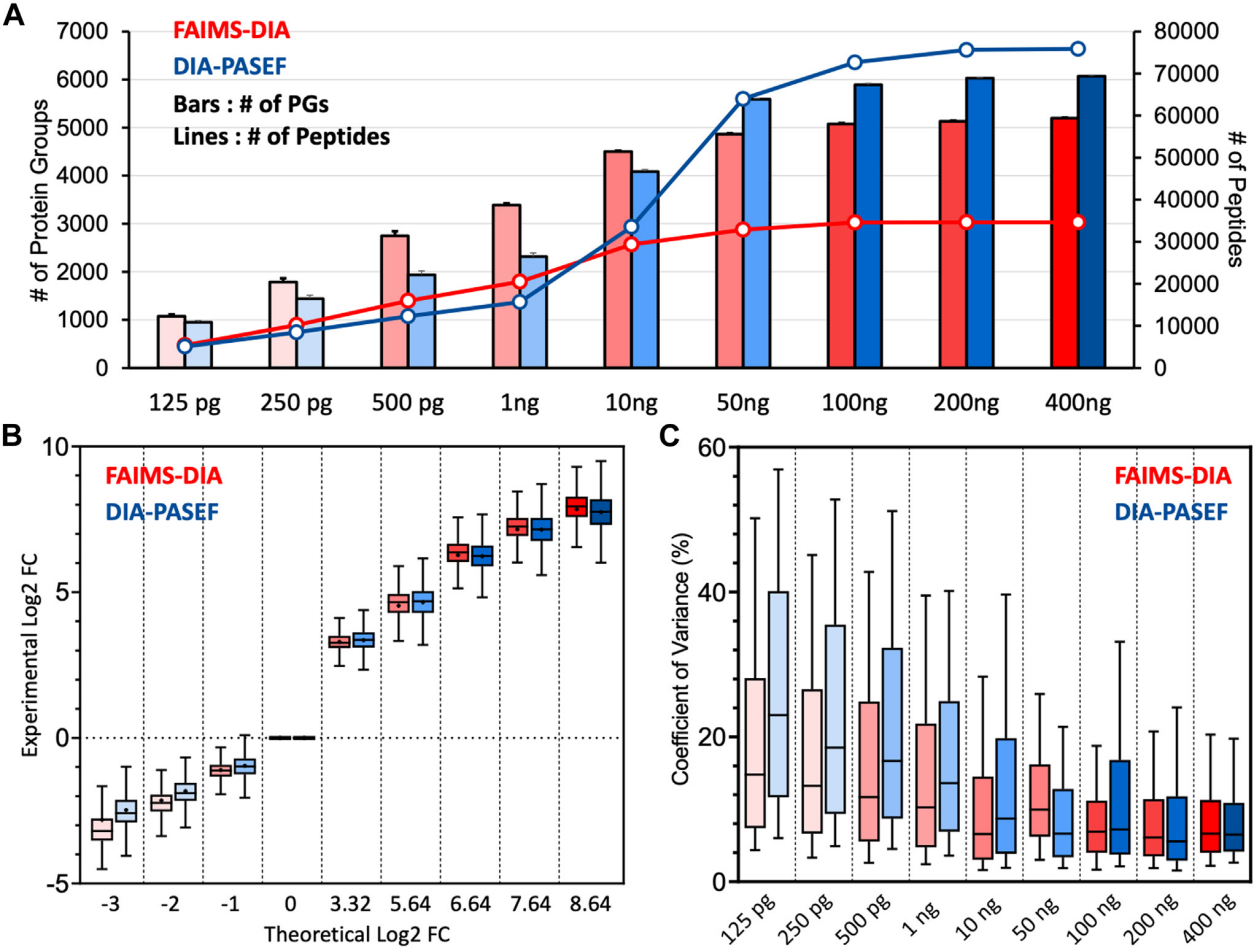
**Analytical performances of DIA-MS in proteomic analysis of single-cell-level peptides with two proteomic platforms**. *A*, Identified peptides and protein groups from serially diluted NCI-7 peptide samples. In this figure, *red* indicates FAIMS-DIA-MS data from the proteomic platform performed on the Orbitrap Ascend setting, and *blue* presents DIA-PASEF data proteomic platform performed on the timsTOF HT setting. *B*, linearity of quantification using the two platforms. Each box plot presents the distribution of Log2 fold-changes average value of protein groups in the sample to the protein group in 1 ng sample. The X-axis shows the theoretical Log2 fold-change we can expect. *C*, reproducibility in each sample per dataset from the two different proteomic platforms.

In our study, we initially assessed identified and quantified proteins from serially diluted peptide samples using two different proteomic platforms: one platform performed by the Orbitrap Ascend and the second one performed on timsTOF HT. The Ascend utilizes FAIMS, which selectively filters ions based on their differential mobility in an oscillating electric field. This selective ion passage significantly reduces chemical noise and boosts the signal-to-noise ratio, which is critical for detecting low-abundance peptides in low-input samples, such as those typical in single-cell proteomics (18). Conversely, the timsTOF HT employs PASEF-DIA, incorporating Tims followed by time-of-flight (TOF) mass spectrometry. timsTOF is effective in achieving the accumulation of ions (Tims) and increased instrument speed and throughput (TOF) (33). Furthermore, the Ascend features Automatic Gain Control (AGC), optimizing the ion count entering the mass spectrometer to ensure stable and consistent signals. In contrast, the timsTOF HT uses ion mobility for sensitive and high-speed analysis. However, both proteomic platforms demonstrated comparable results, as illustrated in Figure 2, *A*–*C*, increased number of proteins were identified and quantified with high-input peptide amount ( ≥ 50 ng) for the proteomic platform on timsTOF and the elevated number of proteins were identified and quantified with low sample input for the proteomic platform on FAIMS-Orbitrap Ascend (Supplemental Table S1 and S2). Based on our evaluation, we decided to use the proteomic platform on FAIMS-DIA for further single-cell analysis.

### Comparison of Proteome Abundance and Cell Volume Between Parental PC3 Cells and DRCs

Given the larger size of DRCs, traditional single-cell isolation methods proved challenging. Initially, we utilized the cellenONE instrument with a recommended cell dilution of 200 cells/μl but frequently found that it resulted in nozzle clogging during cell sorting. To address this, we implemented two modifications: 1) increasing the dilution to 50 cells/μl to reduce cell congestion at the nozzle and allow more space for cells to undergo morphological changes; 2) employing a strainer to filter out the cells clustered together as well as extremely large cells that caused nozzle clogging during cell sorting using cellenONE. Although it was not essential to eliminate the large cells for DRCs studies, this adjustment enabled us to continue working with significantly large DRC cells without issues.

**Fig. 3:**
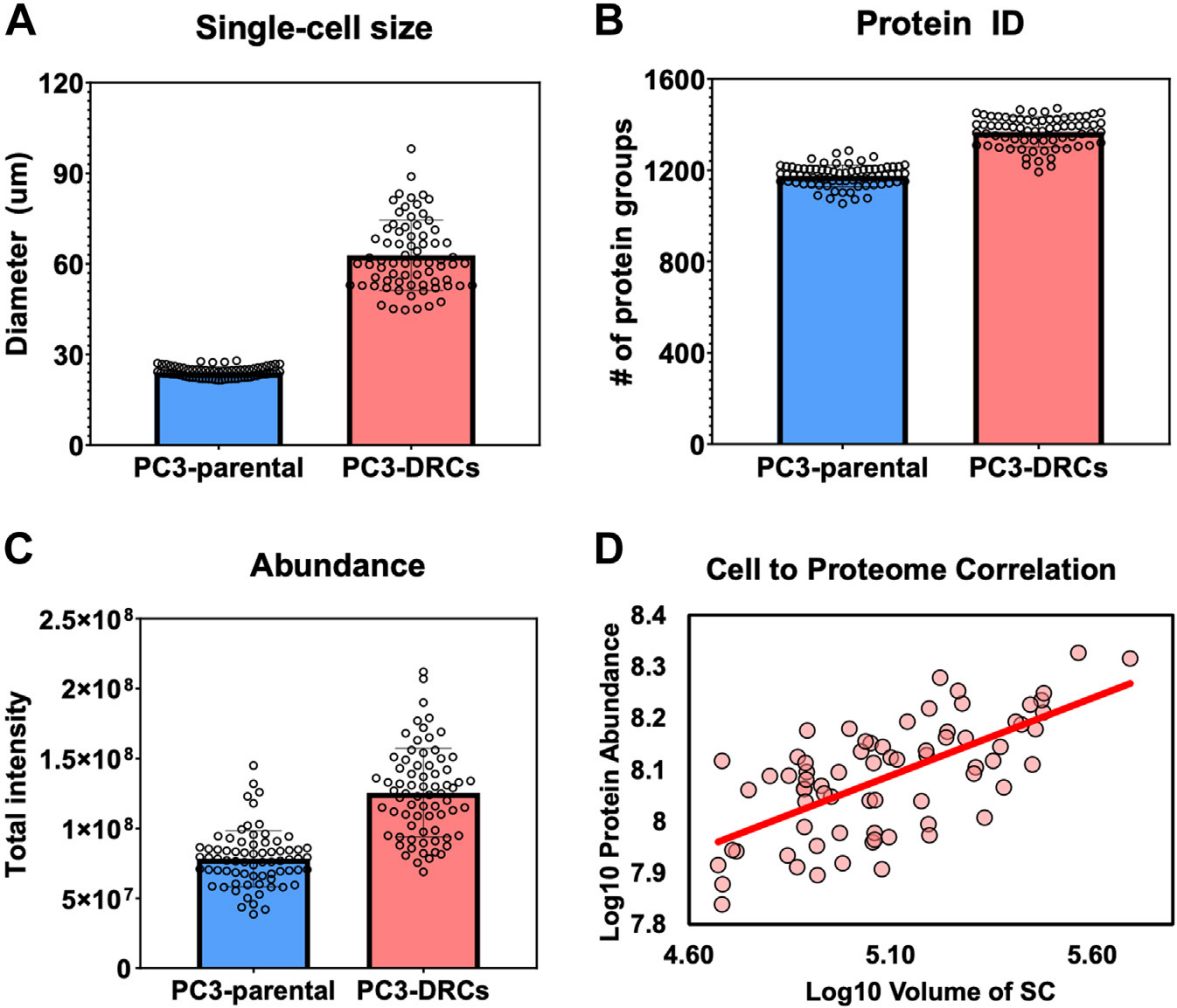
**Comparison of proteome abundance and cell volume between parental PC3 cells and PC3-DRCs**. *A*, cell size distribution in diameter. *B*, identified protein groups per cell. *C*, total protein abundance per cell. *D*, correlation between cell volume (unit: μm^3^) and proteome abundance.

Further analysis of cell size distribution showed marked differences between DRCs and parental PC3 cells. Parental PC3 cells were found to have diameters ranging from 22 to 25 μm, whereas DRCs varied between 45 and 102 μm, demonstrating the substantial morphological changes associated with polyaneuploidy in DRCs (Fig. 3*A*). Proteomic analysis revealed an average of 1174 protein groups per parental PC3 cell and 1367 protein groups per DRC cell, totaling 1567 unique proteins identified across both cell types (Fig. 3*B*, Supplemental Table S3). Quantification of total protein abundance showed significantly higher levels in DRCs compared to parental cells (Fig. 3*C*), correlating with the increased cell volume observed in DRCs. This suggests that DRCs possess a heightened metabolic and biosynthetic capacity to support their larger size and genomic content. A strong positive correlation (Pearson correlation coefficient of 0.69) was found between cell volume and total protein abundance in DRCs, indicating that larger DRCs have the greater proteomic capacity (Fig. 3*D*). This relationship underscores the influence of cell size on the functional capabilities of these cells, highlighting the importance of considering cell volume in proteomic studies of polyaneuploid cells.

### Multidimensional Analysis of Single-Cell Proteome Reveals Distinct Proteomic Landscapes and Subtype Diversity in DRCs of Prostate Cancer

**Fig. 4:**
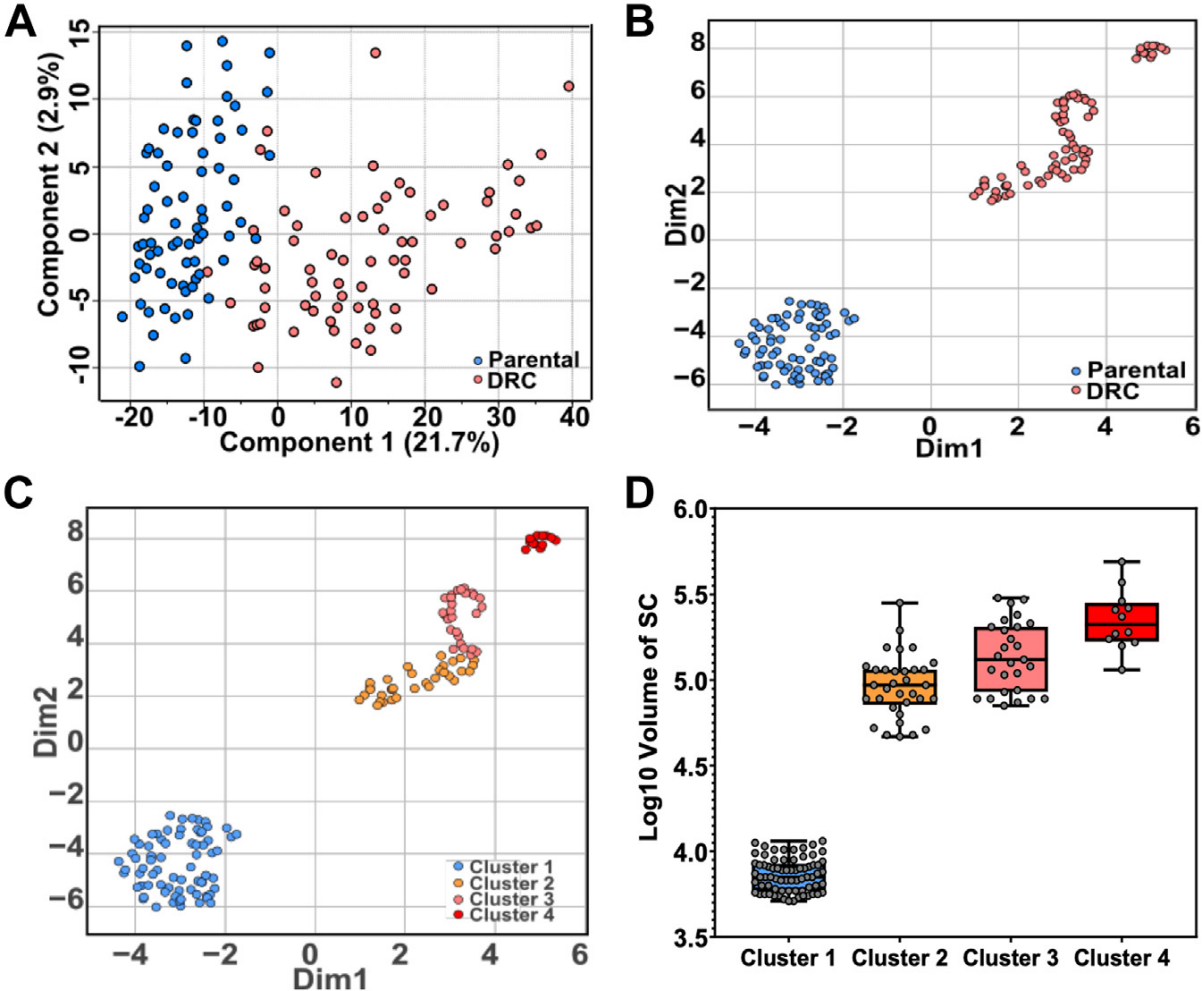
**Multidimensional analysis of single-cell proteome reveals distinct proteomic landscapes and subtype diversity in prostate cancer cells.***A*, PCA plot of 144 single cells. *B*, UMAP - main grouping. *C*, UMAP - Three sub-clusters in DRCs using K-means method. *D*, subgroup clusters and cell size association (unit: μm^3^).

In our single-cell proteomic analysis, incorporating both parental PC3 cells and DRCs, we employed Principal Component Analysis (PCA) and Uniform Manifold Approximation and Projection (UMAP) to explore underlying proteomic diversities and similarities. The PCA results (Fig. 4*A*) revealed a pronounced segregation between the 2 cell types, confirming substantial proteomic disparities. This clear demarcation underscored the significant molecular alterations associated with polyaneuploidy in DRCs, which might contribute to their altered physiological states and therapeutic resistance. Further dissecting the proteomic landscape through UMAP, we not only confirmed the distinct clustering between parental PC3 cells and DRCs (Fig. 4*B*) but also delved deeper into the heterogeneity within the DRCs population. The UMAP analysis, supplemented by k-means clustering, discerned three distinct sub-clusters within the DRCs (Fig. 4*C*, Supplemental Table S4).

Single-cell proteomic analysis of DRCs revealed three distinct clusters—Cluster 2, Cluster 3, and Cluster 4—within the drug-resistant cell population. Each cluster exhibited unique proteomic profiles and functional adaptations to the DNA-damaging agent, as revealed by Reactome pathway enrichment analysis (Supplemental Table S5). Cluster two displayed a strong enrichment in pathways associated with translational and metabolic processes. The most significantly enriched pathways included Translation (37 genes, FDR: 3.35e-18), Metabolism of proteins (87 genes, FDR: 6.39e-17), and Cap-dependent translation initiation (21 genes, FDR: 1.14e-12). Proteins such as H2BK1, EIF2S1, and LDHA were prominently upregulated, highlighting the cluster’s reliance on enhanced protein synthesis and metabolic homeostasis to maintain cellular integrity under stress. Additional enriched pathways, such as Metabolism of amino acids and derivatives (32 genes, FDR: 4.84e-12) and Selenoamino acid metabolism (19 genes, FDR: 4.38e-11), suggest adaptive metabolic rewiring to sustain biosynthetic demands and survival. Cluster 3 exhibited a distinct proteomic profile characterized by stress-response mechanisms and metabolic flexibility. Enriched pathways included Infectious disease (93 genes, FDR: 4.64e-24), Metabolism of RNA (81 genes, FDR: 4.92e-24), and Cellular responses to stress (69 genes, FDR: 6.84e-16). Upregulated proteins, such as HSP90AB1, PARP1, and TFRC, reflect the cluster’s ability to manage proteotoxic stress and maintain metabolic adaptability. Additionally, pathways such as Metabolism of amino acids and derivatives (49 genes, FDR: 1.83e-16) and Translation (50 genes, FDR: 4.12e-21) suggest this cluster’s capacity for biosynthetic and metabolic regulation to support survival and proliferation under therapeutic stress. Cluster 4 was marked by a highly aggressive and invasive phenotype. Enriched pathways included Metabolism of RNA (66 genes, FDR: 2.92e-21), Cellular responses to stress (53 genes, FDR: 2.26e-12), and Metabolism of proteins (103 genes, FDR: 2.14e-17). Upregulated proteins, such as ITGB1, CD47, and PARP1, indicate enhanced migratory capacity and stress resilience. Unique to this cluster, the enrichment of Influenza viral RNA transcription and replication (23 genes, FDR: 1.46e-11) and Influenza infection (26 genes, FDR: 7.20e-13) suggests potential interactions with immune and microenvironmental factors, supporting its invasive behavior. Each sub-cluster represents a unique proteomic signature that potentially corresponds to varying degrees of malignancy and resilience against therapeutic interventions. In addition, the most compelling is the association of these sub-clusters with cell size variations (Fig. 4*D*), where we observed that larger cell sizes correspond to distinct proteomic profiles. This correlation suggests that cell size, a proxy for cellular complexity and metabolic capacity, might be a critical determinant in the phenotypic and functional heterogeneity observed within DRCs.

### Single-Cell Proteomic Characterization Reveals Downregulation of Key Regulatory Proteins in DRCs

**Fig. 5:**
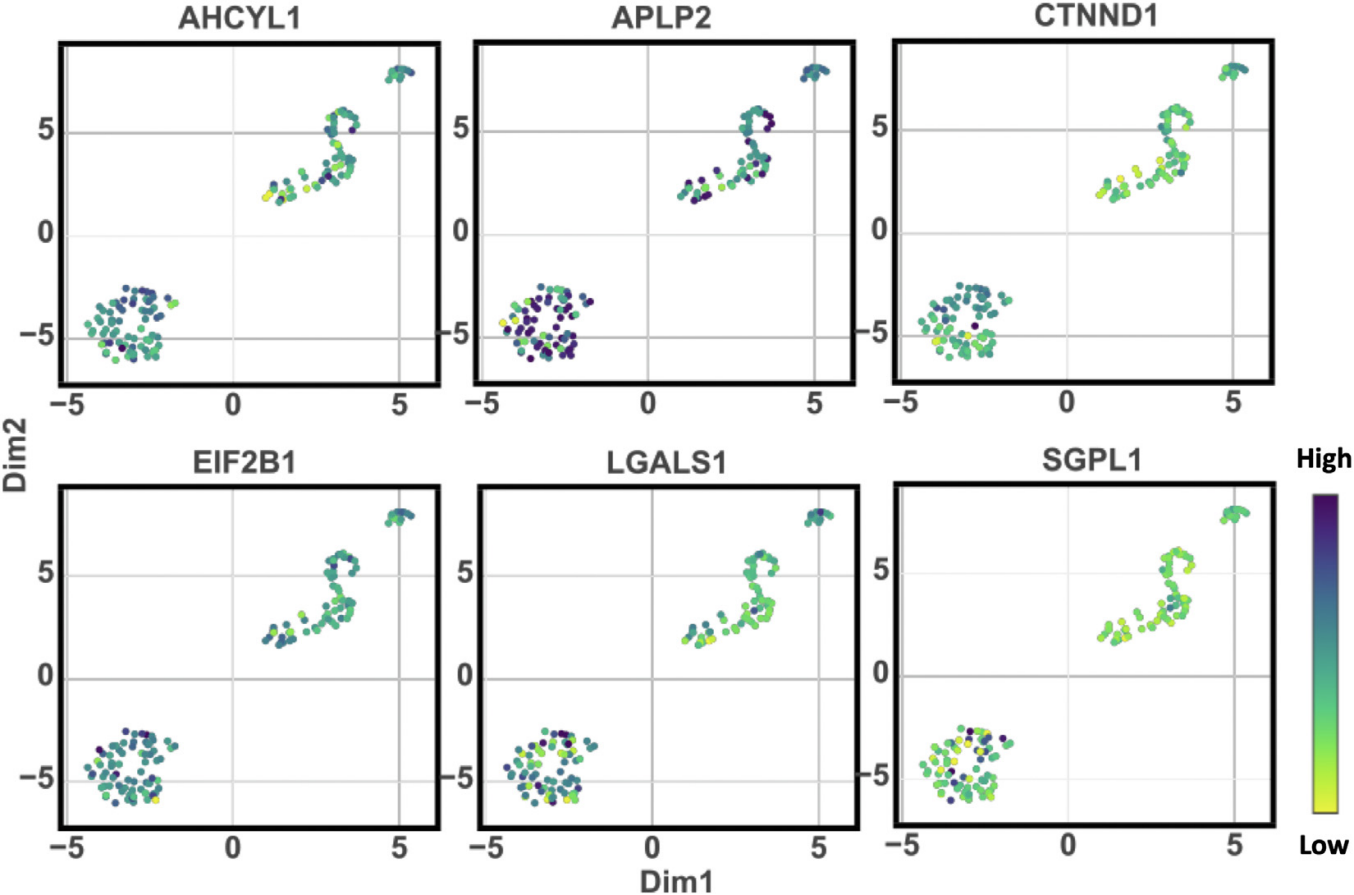
**Downregulated proteins in drug-resistant prostate cells.** Significant down-regulated proteins in DRCs compared to parental PC3 cells (*t* test *p*-value <0.05) including AHCYL1, APLP2, CTNN1D, EIF2B1, LGALS1, and SGPL1 were observed.

In our detailed single-cell proteomic analysis, we identified a significant down-regulation of several proteins in DRCs compared to parental PC3 cells, which could be crucial in understanding the altered physiological states associated with cancer progression and therapy resistance (Fig. 5, Supplemental Table S6). These proteins, including AHCYL1, APLP2, CTNN1D, EIF2B1, LGALS1, and SGPL1, play vital roles in maintaining cellular functions such as cell adhesion, protein synthesis, and lipid metabolism, which are often perturbed in cancer cells. AHCYL1 (S-adenosylhomocysteine hydrolase-like protein 1) is implicated in the regulation of intracellular homocysteine levels and methylation processes, influencing cell plasticity and potentially tumorigenesis (34); its down-regulation could disrupt these critical metabolic processes, contributing to the aggressive traits of DRCs. APLP2 (Amyloid beta precursor-like protein 2), known for its involvement in synaptic function and cellular adhesion (35), might affect cell-cell interaction and migration dynamics when downregulated, potentially facilitating cancer cell detachment and metastasis. CTNN1D (Catenin delta-1), a part of the cadherin-catenin complex, plays a significant role in maintaining cell adhesion (36); reduced levels may lead to decreased adhesiveness and increased metastatic potential. EIF2B1 (Eukaryotic translation initiation factor 2B) is essential for the initiation of protein synthesis (37); its reduction could lead to a broad decrease in protein production, affecting various growth and survival pathways in cancer cells. LGALS1 (Galectin-1) functions in cell-matrix interactions and immune response modulation (38); its downregulation could impact tumor immune evasion and cell communication. SGPL1 (Sphingosine-1-phosphate lyase 1) is critical in sphingolipid metabolism, which is pivotal in regulating apoptosis and cell migration (39); alterations in its levels could modify the balance between cell death and survival, influencing disease progression. The collective downregulation of these proteins in DRCs underscores a strategic alteration in cellular regulatory mechanisms that could confer survival advantages under therapeutic stress or contribute to an enhanced metastatic capacity. Understanding these protein changes provides valuable insights into the complex molecular dynamics that drive cancer cell adaptation and survival, highlighting potential targets for therapeutic intervention aimed at mitigating the aggressive behaviors of DRCs.

### Elevated Levels of Surface Proteins and Transcription Factors Underlines DRCs' Aggressive Traits

**Fig. 6:**
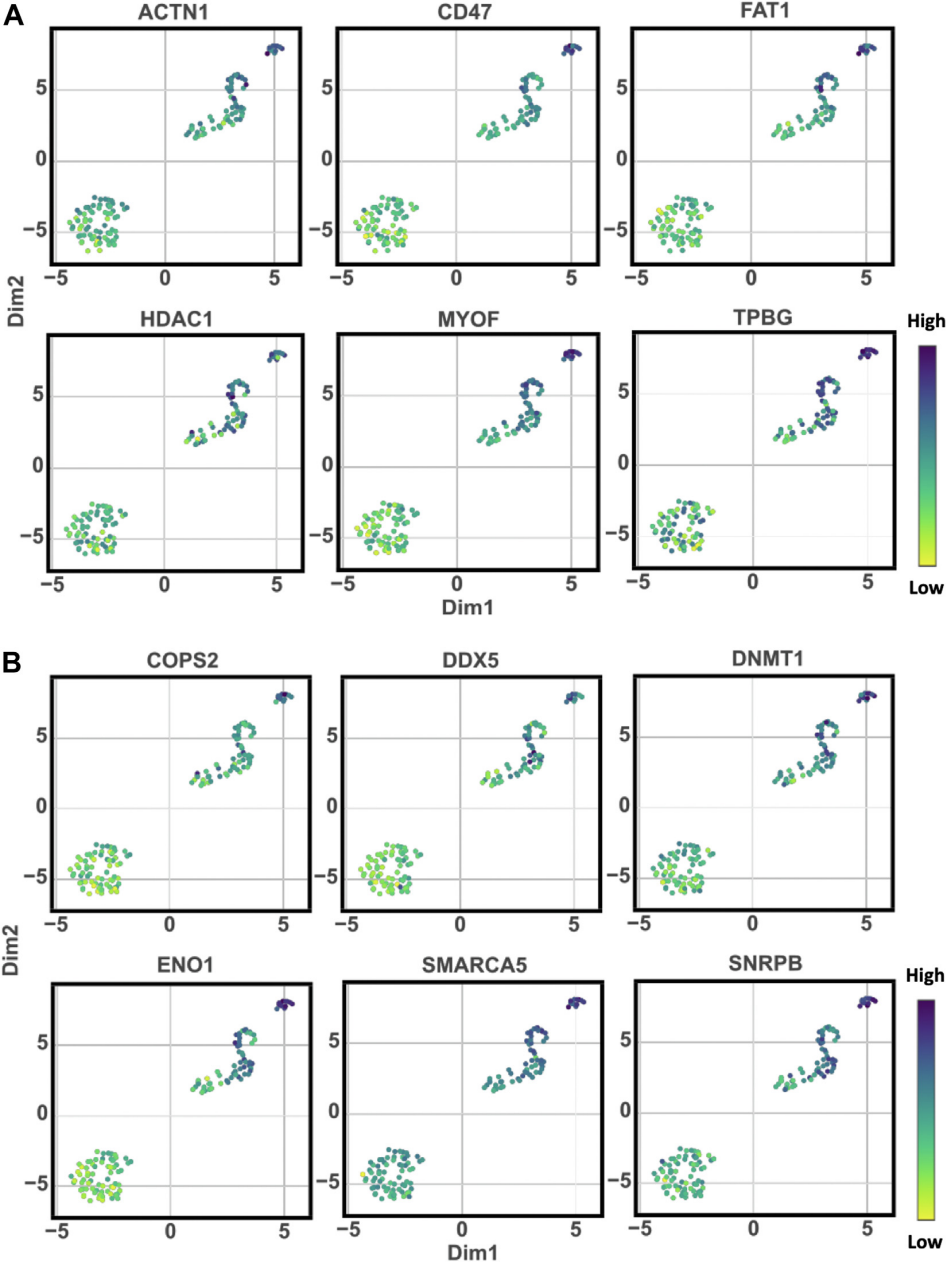
**Significantly up-regulated proteins in DRCs (*t* test *p*-value <0.05)**. *A*, key surface plasma membrane proteins were selected by using the cell surface protein atlas (http://wlab.ethz.ch/cspa/). *B*, transcription factors were taken by the list (49).

In our analysis, we identified a significant up-regulation of key surface plasma membrane proteins and transcription factors in DRCs, which are likely to play crucial roles in promoting the aggressive behavior and resilience of these cancer cells (Supplemental Table S6). Among the surface proteins (Fig. 6*A*), ACTN1 (Alpha-actinin-1), CD47, and FAT1 (FAT atypical cadherin 1) were notably increased. ACTN1, which plays a pivotal role in cell motility and structural integrity (40), might enhance the migratory and invasive capabilities of DRCs. CD47, often referred to as the "don't eat me" signal (41), prevents phagocytosis by immune cells, and its elevated levels suggest a strategic adaptation by DRCs to evade immune surveillance, thereby enhancing their survival in the circulatory system and metastatic niches. FAT1 is involved in modulating cell adhesion and signaling networks (42, 43); its upregulation potentially facilitates more robust cell-cell interactions and could promote the formation of invasive structures.

Furthermore, the elevated levels of transcription factors identified, including COPS2, DDX5 (DEAD-box helicase 5), DNMT1 (DNA methyltransferase 1), ENO1, SMARCA5, and SNRPB, highlight a dynamic alteration in gene expression regulation and epigenetic modifications within DRCs (Fig. 6*B*). These factors are integral to various cellular processes such as RNA processing, chromatin remodeling, and gene silencing. For instance, DDX5 is crucial for the processing of RNA, influencing various aspects of mRNA splicing and ribosome biogenesis (44), which are essential for rapid protein synthesis in fast-growing cancer cells. DNMT1's role in maintaining DNA methylation patterns is critical for the epigenetic regulation of gene expression (45), suggesting that its upregulation could support aberrant cancer cell proliferation by silencing tumor suppressor genes and activating oncogenic pathways.

The collective upregulation of these proteins and factors in DRCs suggests a complex reprogramming of cellular functions that not only enhances the aggressive characteristics of these cells, such as invasiveness and immune evasion, but also supports a robust infrastructure for sustained growth and survival under adverse conditions. This adaptation likely contributes to the formidable challenge DRCs present in clinical settings, underscoring the need for targeted therapies that can disrupt these specific molecular mechanisms.

### Comparison of SCP and scRNA-Seq in Drug-Resistant Cells

**Fig. 7:**
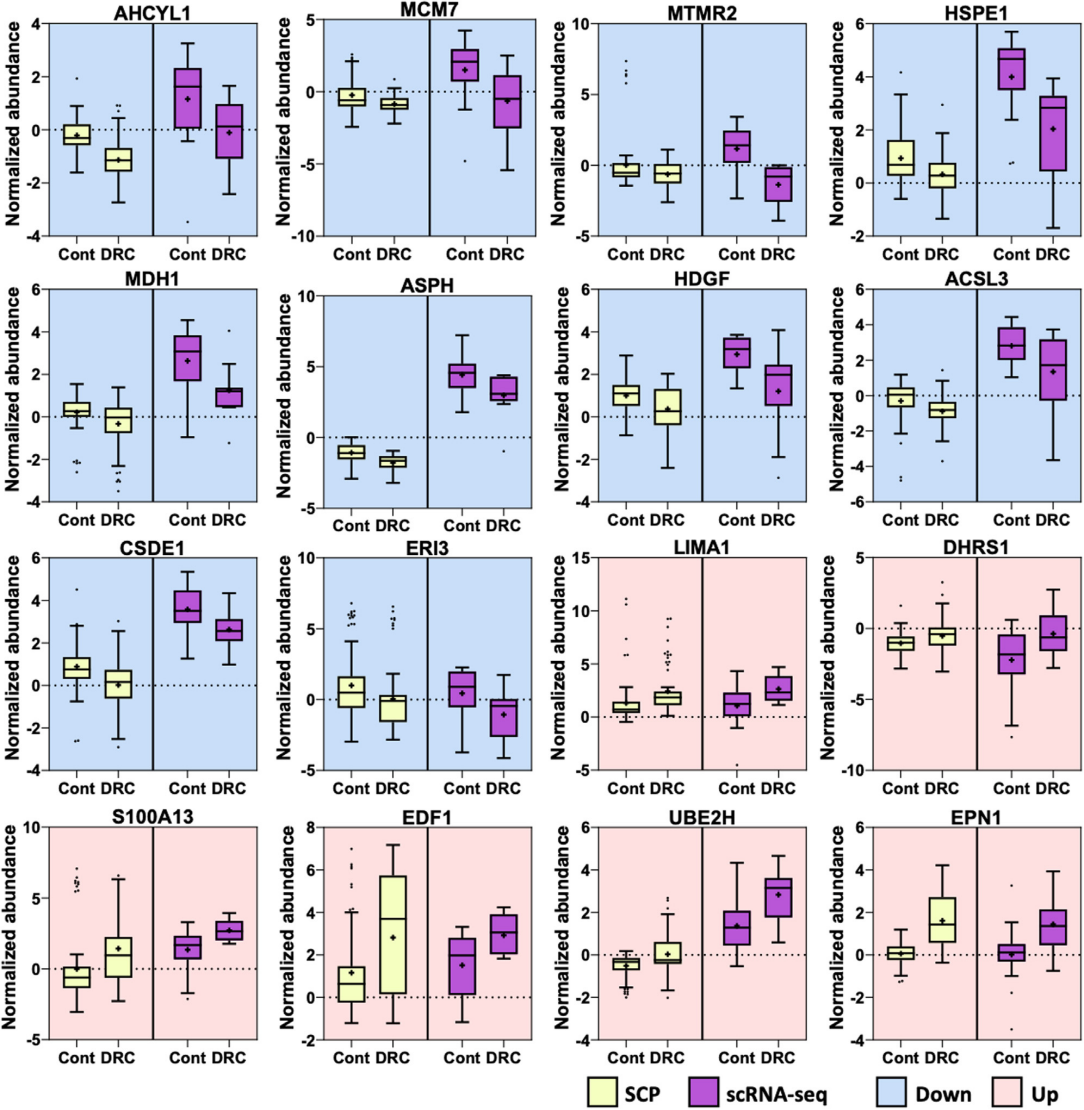
**Genes and proteins significantly altered in both single-cell proteomics (SCP) and scRNA-seq datasets**. The box plots represent significantly regulated genes/proteins commonly identified in both SCP and scRNA-seq analyses, with a *t* test *p*-value <0.05. The purple boxes correspond to the gene expression data, while the *yellow* boxes represent protein expression data. Each gene/protein pair is displayed side by side, illustrating the congruence between transcriptomic and proteomic regulation across drug-resistant and parental cell lines.

To further understand the molecular changes in drug-treated cells, we compared the results of single-cell proteomics (SCP) with RNA-seq data (Supplemental Tables S7 and S8). This integrative analysis revealed consistent changes across both omics platforms, providing a comprehensive view of gene expression and protein abundance alterations in drug-treated cells. We identified six genes that were upregulated and 10 genes that were downregulated in both the SCP and RNA-seq datasets (Fig. 7), indicating a robust regulatory pattern in drug-treated cells. LIMA1, DHRS1, S100A13, EDF1, UBE2H, and EPN1 showed increased expression levels at both the transcriptomic and proteomic levels, suggesting these molecules play a crucial role in the cellular adaptation to drug resistance. Notably, LIMA1 (LIM domain and actin-binding protein 1) is involved in actin cytoskeleton organization, which may contribute to the enhanced motility and invasiveness of drug-resistant cells. Similarly, S100A13 is associated with stress responses and extracellular signaling, further underscoring the aggressive nature of these cells. MCM7, MTMR2, HSPE1, MDH1, ASPH, HDGF, ACSL3, CSDE1, ERI3, and AHCYL1 were significantly downregulated in both omics datasets. MCM7, a member of the minichromosome maintenance complex, plays a critical role in DNA replication initiation, and its downregulation may suggest a slower replication cycle in drug-treated cells. The downregulation of proteins involved in metabolic processes, such as MDH1 (malate dehydrogenase 1) and ASPH (aspartate-β-hydroxylase), could indicate alterations in the energy production pathways, which may contribute to the survival of these cells under drug stress.

## Discussion

An important aspect of this study was the evaluation of two different proteomic platforms for single-cell proteomics by DIA-MS using the timsTOF HT and the FAIMS Orbitrap Ascend. Both proteomic platforms were tested for their capability to identify and quantify proteins at the single-cell level, and their performance was compared based on factors such as sensitivity, reproducibility, and depth of coverage. While both instruments demonstrated strong performance, we ultimately chose the Orbitrap Ascend coupled with FAIMS for our single-cell proteomic analysis. The decision was based on the superior ability of this instrument to handle low-input samples, its enhanced signal-to-noise ratio, and its consistent detection of low-abundance proteins. The FAIMS feature of the Orbitrap Ascend significantly reduced chemical noise and improved the detection of peptides, making it particularly advantageous for single-cell proteomics where sample input is limited. This consistency was critical in identifying distinct proteomic sub-clusters within the drug-resistant cell (DRC) population, which may have been more challenging to detect with other MS platforms.

The single-cell proteomic analysis of drug induced DRCs and the parental prostate cancer cells revealed a substantial increase in both the number of identified proteins and total protein abundance in DRCs compared to parental PC3 cells. The strong correlation between cell volume and proteome abundance underscores the importance of cell size in understanding the proteomic capacity and functionality of DRCs. Larger DRCs, with their increased protein content, may have higher metabolic and biosynthetic activity, contributing to their survival and adaptability under therapeutic stress.

An unsupervised clustering analysis demonstrated clear proteomic distinctions between parental PC3 cells and DRCs, with DRCs further subdivided into three sub-clusters. This intra-population heterogeneity, associated with cell size, suggests that DRCs may comprise multiple subtypes with potentially different biological characterization. Such size-related diversity could have profound implications for understanding the biology of cancer cell survival, proliferation, and metastasis, especially in the context of therapeutic resistance. These insights not only emphasize the necessity for single-cell resolution in studying cancer heterogeneity but also highlight how advanced proteomic profiling can illuminate the complex interplay between cellular morphology and molecular function. This enhanced understanding could pave the way for more targeted and effective therapeutic strategies that consider both the molecular and morphological diversity within tumorous cell populations. Understanding these sub-clusters' specific characteristics could inform the development of targeted therapies aimed at exploiting the unique weaknesses of each DRC subtype.

A notable observation in our study is the low correlation (Pearson r = 0.21) between the scRNA-seq and scProteomics datasets, consistent with previous reports indicating weak concordance between mRNA and protein abundance across various biological systems (15, 46, 47). This discrepancy can be attributed to the complex regulatory mechanisms that govern by regulation of gene expression, control of protein translation, protein post-transcriptional modifications, and protein degradation, which are not captured at the RNA level. For instance, we observed key differences, particularly in surface proteins and transcription factors. Several plasma membrane proteins, including CD47, ACTN1, and FAT1, showed elevated levels in drug-treated cells at the protein level, yet their mRNA levels were not detected or weakly expressed in the RNA-seq dataset. Similarly, transcription factors such as DDX5, DNMT1, and SMARCA5 exhibited increased protein abundance in drug-treated cells but had limited corresponding mRNA changes. These findings emphasize the value of proteomics in capturing the functional state of cells, as it directly measures the proteins that drive cellular behavior, therapeutic resistance, and disease progression. While transcriptomic analysis provides valuable insights into gene expression programs, proteomics reveals the actual molecular players responsible for executing cellular functions. Additionally, the intra-method correlation was significantly higher within scProteomics data (Pearson r = 0.78) compared to scRNA-seq data (Pearson r = 0.55), suggesting that single cell proteomic measurements show considerable consistency when analyzing functional states of cells. This aligns with prior studies demonstrating that proteomics more accurately reflects cellular phenotypes and functional changes compared to transcriptomics (48). These findings highlight the unique strengths of single-cell proteomics in providing deeper insights into the molecular mechanisms underlying drug resistance, especially in cases where transcriptomic data alone may be insufficient to capture functional heterogeneity.

The identification of unique molecular signatures in DRCs provides insights into the pathways and processes that differentiate them from parental PC3 cells. Downregulated proteins such as AHCYL1, APLP2, and CTNN1D in DRCs may indicate the suppression of pathways involved in normal cellular functions, impacting cell adhesion and protein synthesis. Conversely, up-regulated surface plasma membrane proteins like ACTN1, CD47, and FAT1 suggest enhanced pathways related to cell structure, immune evasion, and cell adhesion. Additionally, transcription factors such as COPS2, DDX5, and DNMT1 were upregulated, indicating potential changes in gene expression regulation that may drive the unique characteristics of DRCs. The integration of SCP and RNA-seq data provided a robust validation of these changes at both the proteomic and transcriptomic levels. These proteomic changes, in combination with the transcriptomic data, suggest that drug-treated prostate cancer cells employ a dual strategy for survival: increasing their cellular flexibility and invasive potential while downregulating key metabolic and proliferative pathways. This reprogramming enables them to withstand therapeutic pressure and may explain their enhanced ability to evade chemotherapy and establish metastases. Targeting these altered pathways, particularly those involved in cytoskeletal dynamics, immune evasion (e.g., CD47), and metabolism, may provide new opportunities for therapeutic intervention. In addition, the identification of three distinct clusters within the drug-resistant cell population highlights the functional heterogeneity contributing to therapeutic resistance. Cluster two demonstrated a reliance on translational and metabolic stability, supported by the upregulation of proteins such as H2BK1, EIF2S1, and LDHA, which enable the maintenance of protein synthesis and cellular recovery under stress. Cluster three was characterized by robust stress-response mechanisms and metabolic flexibility, with proteins such as HSP90AB1, PARP1, and TFRC driving its capacity to adapt to proteotoxic and metabolic challenges, ensuring survival under adverse conditions. In contrast, Cluster 4 exhibited a highly invasive and aggressive phenotype, driven by proteins such as ITGB1, CD47, and PARP1, which enhance migratory capacity, immune evasion, and proliferative potential. These findings emphasize the distinct survival strategies of each cluster, with Cluster two focused on translational homeostasis, Cluster 3 prioritizing stress adaptation, and Cluster four driving invasion and metastasis. This study underscores the importance of targeting subpopulation-specific vulnerabilities to overcome resistance and improve therapeutic outcomes.

This study's findings highlight the potential of single-cell proteomics in providing detailed molecular insights into the heterogeneity and complexity of cancer cells. By combining these detailed proteomic insights with advanced single-cell analysis techniques, we aim to better understand the molecular underpinnings of drug-resistant cancer cell behavior and develop targeted therapeutic strategies to combat their aggressive and resistant nature. This study serves as a foundational step towards more personalized and effective cancer treatments, highlighting the critical role of single-cell proteomics in cancer research.

## Data Availability

The mass spectrometry proteomics data have been deposited to the ProteomeXchange Consortium (50) via the PRIDE partner repository with the dataset identifier PXD059079.

## Supplemental Data

This article contains supplemental data.

## Conflict of Interest

The authors declare the following financial interests/personal relationships which may be considered as potential competing interests: K.J.P. discloses serving as a consultant for Cue Biopharma, Inc., holding equity in PEEL Therapeutics, and being both a founder and equity holder in Keystone Biopharma, Inc. and Kreftect, Inc. S.R.A. discloses holding equity in Keystone Biopharma, Inc. H.Z. discloses serving as a co-founder of Complete Omics Inc. The remaining authors have no conflicts of interest to declare.
